# Frequency of repositioning for preventing pressure ulcers in patients hospitalized in ICU: protocol of a cluster randomized controlled trial

**DOI:** 10.1186/s12912-021-00616-0

**Published:** 2021-07-05

**Authors:** Olga L. Cortés, Mauricio Herrera-Galindo, Juan Carlos Villar, Yudi A. Rojas, María del Pilar Paipa, Luzdary Salazar

**Affiliations:** 1 Research Unit & Nursing Department, Fundación Cardioinfantil-Instituto de Cardiología, Cl. 163a #13B-60, Bogotá D. C, Colombia; 2 Faculty of Health Sciences, Universidad Autónoma de Bucaramanga, Bucaramanga, Colombia Avenida 42 No 48-11PBX,; 3 Nursing Department, Fundación Cardioinfantil-Instituto de Cardiología, Bogotá D. C, Cl. 163a #13B-60, Colombia; 4 Intensive Care Units, Nursing Department, Fundación Cardioinfantil-Instituto de Cardiología, Bogotá D. C, Cl. 163a #13B-60, Colombia; 5 Ambulatory Nursing Department, Fundación Cardioisnfantil-Instituto de Cardiología, Bogotá D. C, Cl. 163a #13B-60, Colombia

**Keywords:** Critical care, Pressure ulcers, Bedsores, Nursing, Randomized, Clinical trial, Clusters

## Abstract

**Background:**

Despite being considered preventable, ulcers due to pressure affect between 30 and 50% of patients at high and very high risk and susceptibility, especially those hospitalized under critical care. Despite a lack of evidence over the efficacy in prevention against ulcers due to pressure, hourly repositioning in critical care as an intervention is used with more or less frequency to alleviate pressure on patients’ tissues. This brings up the objective of our study, which is to evaluate the efficacy in prevention of ulcers due to pressure acquired during hospitalization, specifically regarding two frequency levels of repositioning or manual posture switching in adults hospitalized in different intensive care units in different Colombian hospitals.

**Methods:**

A nurse-applied cluster randomized controlled trial of parallel groups (two branches), in which 22 eligible ICUs (each consisting of 150 patients), will be randomized to a high-frequency level repositioning intervention or to a conventional care (control group). Patients will be followed until their exit from each cluster. The primary result of this study is originated by regarding pressure ulcers using clusters (number of first ulcers per patient, at the early stage of progression, first one acquired after admission for 1000 days). The secondary results include evaluating the risk index on the patients’ level (Hazard ratio, 95% IC) and a description of repositioning complications. Two interim analyses will be performed through the course of this study. A statistical difference between the groups < 0.05 in the main outcome, the progression of ulcers due to pressure (best or worst outcome in the experimental group), will determine whether the study should be put to a halt/determine the termination of the study.

**Conclusion:**

This study is innovative in its use of clusters to advance knowledge of the impact of repositioning as a prevention strategy against the appearance of ulcers caused by pressure in critical care patients. The resulting recommendations of this study can be used for future clinical practice guidelines in prevention and safety for patients at risk.

**Trial registration:**

PENFUP phase-2 was Registered in Clinicaltrials.gov (NCT04604665) in October 2020.

## Background

Pressure ulcers (PU) are wounds that affect the skin, caused by prolonged contact or friction in certain points where increased mechanical pressure is applied by the patients’ own weight, especially under skeletal prominence areas (such as the sacrum, trochanters, scapulae, or shoulder blades, heels, and elbows) [1–3]. An increased rate of these events and their complications determine changes in the physical and mental state of the patients and their families, a decreased quality of life level, in addition to higher hospital costs and an increase in healthcare system expenses [1].

The absence of pressure ulcers is considered a quality indicator for installed safety programs and special care given to patients at hospitalization-level risk; therefore, prevention plans for these events should be implemented in patients at a higher risk. Even though considered preventable, PUs constitute between 10 and 50% of adverse events while affecting between 30 and 50% of patients at high and very high risk, especially those hospitalized in critical care services [2, 4]. The incidence of PUs in patients admitted into ICUs varies from 3,3 and 52,9% worldwide [5]. .These patients suffer a diminishing of their natural perception due to the effects of sedation and analgesia required for these services. As a result, a chronic and painful pressure over any area of the body produces little to no reaction in patients for them to change their position.

Among the different factors associated with the emergence of PUs, there is age (OR 2.14 [95% IC 1.27–3.62]), diabetes (OR 5.58 [95% IC 1.58–18.7), duration of MBP < 60-70 mmHg (OR 1.09 [95% IC 1.02–1.17]), mechanical ventilation (OR 23 [95% IC 6.42–86.6]), continuous venovenous hemofiltration or intermittent dialysis (OR 3.7 [IC 95% 1.03–13.86]), use of vasoactive drugs (OR 1.02[95% IC 1.02–1.03]), use of sedatives (OR 1.02 [95% IC 1.01–1.03]) and a low amount of shifts in position (OR 3.63 [95% IC 1.09–12.05]). The information proved that wounds due to pressure and pressure ulcers occur more frequently in patients who are hardly mobilized at lower rates in 24 h (OR 3.63 IC 95% [1.09–12.0]), meaning a repositioning rate of 4 times in 24 h [5, 6]; and a likelihood of 2,96 for grade II pressure ulcers (95% IC 1.23–7.15) [6].

.Within diverse clinical practices for preventing PUs and presenting a higher efficacy, there are anti-decubitus mattresses, the use of gels in surgical patients, genital care in patients with incontinence, and foam bandages in the sacrum region [2, 6]. However, despite its low evidence and no efficacy results, repositioning is also recommended as a prevention strategy.

Repositioning is a nursing care practice utilized for preventing PUs, and its main objective is to reduce mechanical pressure on the skin (which is increased by gravity force) in the aforementioned areas at risk, which is under skeletal prominence areas in patients who completely motionless and in bed rest [7, 8]. Information regarding the impact of repositioning frequency in patients in ICUs has been indirectly obtained from cohort studies and systematic revisions of related literature [9–15].

These studies have reported that the emergence of second and first-degree PUs occurs when a static patient is repositioned at very low rates. This is up to 6 times a day (OR 3.63 IC 95% 1.09–12.0) [5, 6]. However, the precise frequency of repositioning, which would best reduce these wounds’ emergence, remains unknown. The existing evidence reported from systematic revisions of literature on posture shifting’s impact showed a great limitation in pre-existing studies, mainly due to a lack of means and precision to reach valid results [7].

Clinical Practice Guidelines indicate that a patient with an alteration in their state of consciousness should be mobilized frequently every 2 h and that mobilization is better than no mobilization whatsoever [1, 6]. However, levels of information on this recommendation are low according to a Grade evaluation [1, 6]. This implies a lack of evidence on the impact of repositioning in the prevention of PUs.

In addition to this, there is no evidence of possible complications associated with different mobilization frequencies [7]. Studies on routinely preventive interventions, such as the postural shifts and their efficacy in care for patients at high risk for ulcers in ICUs, deserve to be revised carefully. This study aims to solve any uncertainty that exists over what repositioning frequency in static patients best reduces PUs and the possible complications of such interventions. We have planned to perform a pressure ulcer prevention study by nursing in its second phase (PENFUP phase 2). We hypothesize that more frequent repositioning (≤ to every 2 h) performed by nursing staff and critical patients is more effective in reducing the development of pressure ulcers than any other conventional repositioning (applied less frequently ≥ to every 4 h).

Therefore, we have developed a cluster randomized controlled clinical trial that is also multi-centric, pragmatic, and double-blind, made up of two branches comparing a frequent repositioning intervention of 2 h or less with a group of conventional care.

The specific aim is to evaluate the efficacy in preventing PUs acquired during hospitalization on two frequency levels of repositioning or posture shifts in adult patients in different ICUs (clusters) in different regions across Colombia. The three specific aims of this study are 1) to evaluate the efficacy in the intervention of greater frequency, relative to the group of conventional care in the emergence of pressure ulcers and secondary outcomes by clusters; 2) to analyze the hazard ratio (HR) of patients included in the repositioning group of greater frequency compared to the group of patients included in the conventional group in the development of their first PU during their stay in ICUs, and 3) to describe complications related to changes in the position of patients in different ICUs of different hospitals across Colombia.

## Methods

The protocol for this clinical trial is reported according to the SPIRIT guidelines/methodology (http://www.spirit-statement.org/wp-content/uploads/2013/01/SPIRIT-Checklist-download-8Jan13.pdf). The study was registered at Clinicaltrials.gov under registration number NCT04604665 on October 26, 2020 (Not recruiting yet, Version No 1).

### Study design

The “Pressure ulcer prevention by nursing (PENFUP phase II) study” is a pragmatic cluster randomized controlled clinical trial of parallel groups, multicenter, with a blind evaluation of outcomes for data analysts [16]. The study compares the impact of a high-frequency indication of repositioning (every 2 h) in developing pressure ulcers with conventional care in adult- intensive care units (ICU).

**Fig. 1:**
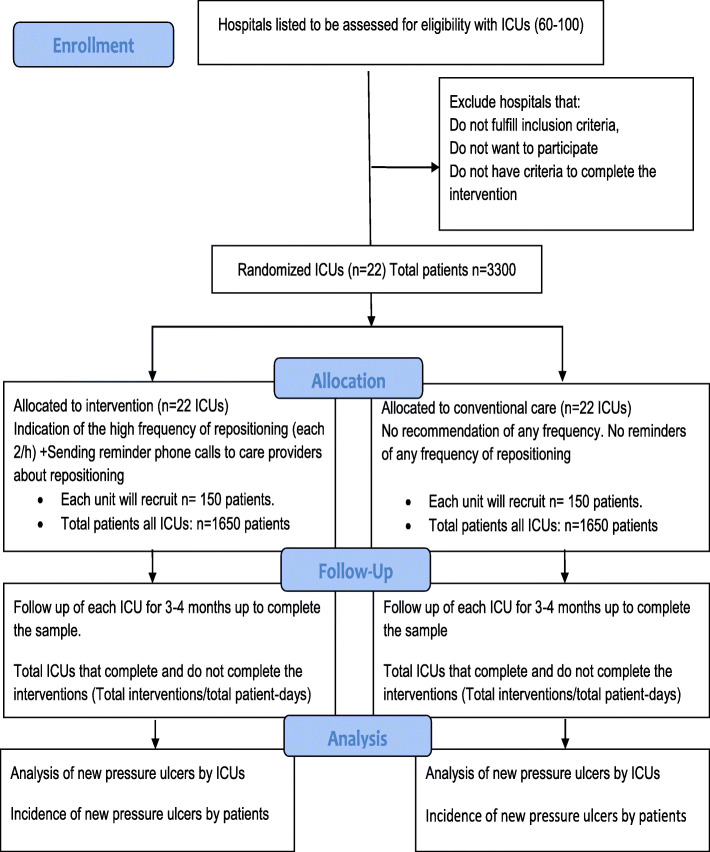
Consort flow diagram

Eligible intensive care units from hospitals of Colombia will be randomized and allocated to perform interventions in which staff from each ICU will be in charge of applying postural shifts (repositioning) at a high frequency that is lesser than or equal to every 2 h in 24 h’ span (experimental group A) or to perform posture shifts according to conventional care (Fig. 1. PENFUP Phase 2-Flowdiagram).

### Setting

**Fig. 2:**
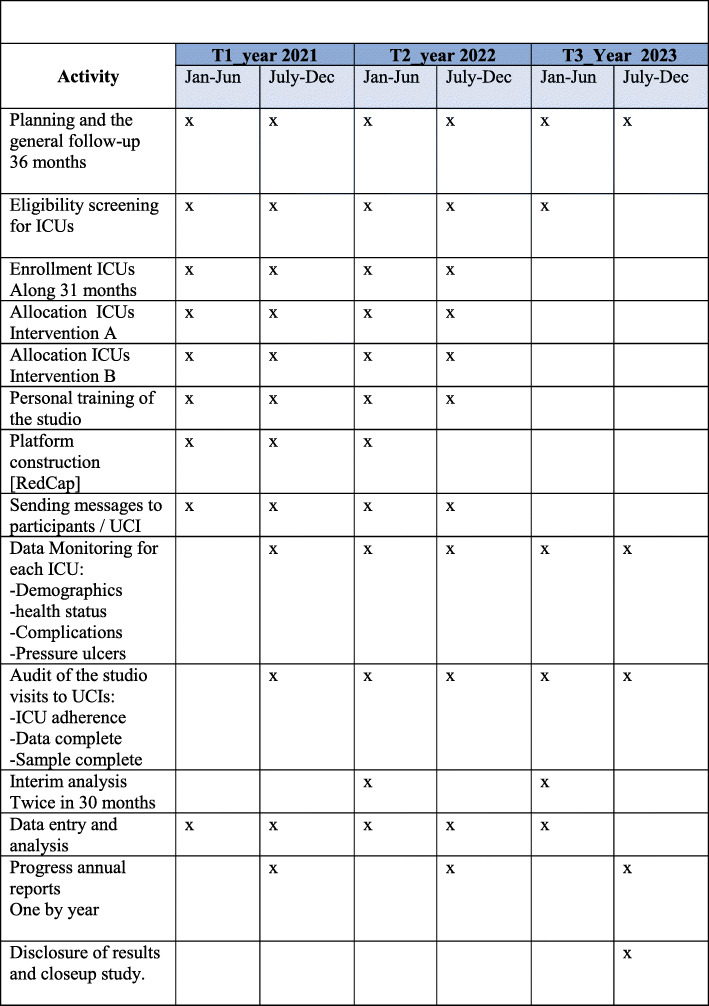
SPIRIT timeline of the study. The complete timeline of the study is about 36 months

All hospitals of various regions of Colombia (north, south, central-east, and west) that have adult ICUs will be asked to partake. Hospitals will be eligible if their heads of staff, including hospital nurse director, chief nurse, and medical director in their ICU’s, accept to participate, in addition to accepting the use of usual practices that would be assigned randomly. The complete study has a duration of 36 months, and the recruitment of patients in each ICU are expected to be completed in a timeline of 3–4 months (Fig. 2).

### Inclusion and exclusion criteria

Criteria for clusters (ICUs) inclusion: ICUs that admit adult patients (≥ 18 years) in critical condition, with any type of care emphasis (surgical, medical, neurological, or a mixture of any), with a minimum of 10 beds, that submit the feasibility survey and agree to commit to the care assigned in the randomization until including the total number of the sample. At least one ICU will be included from each hospital. In case one hospital may participate with more than one ICU, the units should have to receive the same allocation resulting from the randomization.

Criteria for clusters exclusion: intermediate care units or coronary care units in which patients are self-mobilized; or critical units for burned patients. Also, units destined for the care of patients with Covid 19 in which the standard of repositioning is between every 12–16 h.

Criteria for patient inclusion in each cluster: all patients are eligible as long as they are completely unable to move, have their skin intact at the moment of their admission into the ICU, are care-dependent for their complete mobilization (including unconsciousness, being under sedation or assisted ventilation), and are scheduled for a hospitalization period of at least 48 h.

### Sample size

Twenty-two ICUs will be included, each with a total of 150 patients in each branch of the study until completing a sample size of 3.300 patients. The calculation for this sample considered the rate of pressure ulcers observed in PENFUP phase 1 (7.5%) in the control group (patients who received preventative care with skin moisturizing) [17]. As a result of this process, it was decided that the highest incidence rate acceptable for the conventional group for repositioning would be 0.075 and that the incidence in the intervention group with high repositioning frequency would be 0.023 (2.3/7 = 0.32).

For the hypothesis of the study, we calculated a 50% low-impact reduction in pressure ulcers in ICUs that execute repositioning at a high frequency (intervention group A), compared to the ones that implement repositioning at conventional care or control group. The number of ICUs and individuals in there would be obtained by a calculation considering an intra-class correlation coefficient of 0.035 previously attained in the Chaboyer study et al. (2016), a power of 80%, and an alpha error of 0.05% resulting in a 50% difference between both intervention groups [18].

### Randomization and blinding

Previous consent to participate will be solicited for the director of each cluster (medical director of the ICU, nursing coordinator of the ICU) and for nursing staff that will perform the intervention.

All the ICUs participants will be randomized (ratio 1:1) to be assigned to either an intervention group (an indication of repositioning every 2 h) or to conventional care (Intervention provided by care providers using a non-standardized frequency of repositioning). The randomization will be set in the main execution center of the study, which is Fundación Cardioinfantil located in Bogotá, and will be computerized with random numbers. Besides, randomization will be executed using a permutation design by blocks of two possible combinations (intervention A or B). The allocation will be openly announced in each hospital. The study will be blind to evaluators (internal analysts and statisticians) [16, 19, 20]. Furthermore, following this, we plan to apply a consent form for the patients [or their parents according to their health status] when admitted to the ICU.

### Design of Intervention

#### Intervention group a (frequent repositioning ≤ to every 2 h)

The intervention group received a repositioning with a frequency interval which we call “high-frequency” to be applied to every admitted patient consecutively, at a level that is lesser than or equal to every 2 h in a 24/h span (this is with a minimum goal of 8–12 position shifts in 24 h minus 2 o 3 at night to prevent altering the patients’ circadian rhythm). Changes in position will be executed so that patients go from a lateral-right position to a supine position, and later a lateral-left position; or from a lateral-right position to a lateral-left position or vice versa; or from a supine position to a prone position or vice versa [only if this repositioning is provided every 2 h]. The supine position can be overlooked as long as the postural shift is made at the indicated time.

#### Control group B (conventional care)

The units randomized to this care will be labeled as conventional care will not implement any intervention. Patients in this group will receive the usual care for repositioning applied at any frequency.

Furthermore, patients from both groups will receive all other interventions that are currently applied in their hospitals regarding the prevention of PUs according to their hospital’s protocols. Additionally, repositioning will be performed according to routine practices related to initiation, interruption, re-initiation, or finalization of posture changes, depending on the state of health of each patient, medical orders, and nursing decisions. Patients recruited in each ICU will be follow up when they develop their first PU (stage I), or when they begin the autonomous mobilization [ambulation], or when they are transferred to service to another hospital, or they discharge to their homes, or if they die in the ICU.

### Outcomes

Primary outcome: incidence of pressure ulcers by ICU are obtained from the total of PU-bearing individuals, developed during the period of the study in each ICU by each group of clusters. The number of bedsores (Grade I –grade IV) is newly acquired after admission into ICU for 1000 days.

Secondary outcomes: risk of PU (hazard ratio, HR) and time to event individually in patients assigned to the intervention group and the active control group, taking into account the first event of each patient. Also, we will include safety results such as complications due to postural changes (permanent or sporadic shifts in ventilation measures, hypoxemia, hemodynamic changes like, for example, tachycardia or hypertension; events such as severe respiratory insufficiency; cardiogenic shock, pneumonia, or death).

### Classification of pressure ulcers

Pressure Ulcers would be classified as grade I to IV according to The Pressure Ulcer Advisory Panel Consensus Development Conference [21] (NPUAP) 1989, and the National group for the study and counseling in Pressure Ulcers and Chronic Wound-Spain [22] (GNEAUP), 2003. We did not use the new pressure ulcer terminology (pressure injury) and the updated stages provided by The National Pressure Ulcer Advisory Panel- NPUAP, given that all hospitals in our country follow the Ministry of Health Guidelines according to the primary pressure ulcers classification and also Pus are reported in this way.

This system, accepted until very recently, establishes four stages for pressure ulcers. The main objective of any classification system is to standardize the collection of information and provide a standard description of the severity of the ulcer for both clinical practice, evaluation, or research purposes.

This classification defines ulcers from grade I to grade IV. Grade I describes an ulcer with erythema that does not pale on intact skin. Edema, induration, discoloration, and local heat are seen in dark-skinned patients. Stage II is described as a partial skin loss that affects the epidermis, dermis, or both. It is classified as a superficial ulcer with the appearance of abrasion, blister, or shallow crater. Grade III ulcer is described as a total loss of skin thickness that involves injury or necrosis of the subcutaneous tissue, which may extend downward but not through the underlying fascia. Stage IV is considered the total loss of thickness with extensive destruction, tissue necrosis, or injury to muscle, bone, or supporting structure. In this stage, lesions such as caverns, tunnels, or winding paths can occur. This classification is the currently way each hospital has to report PU to the Ministry of Health and Social Protection in Colombia.

### Data collection and procedure

Various hospitals from different regions of Colombia will be recruited from a list of 60–100, each of which will be handed a feasibility survey and a prior discussion, which will help decide each hospital’s eligibility for entering the study. Any hospital that decides that the study is allowed to proceed in its facilities (depending on board matters, nurse-patient relationship, and prior consent to execute interventions resulting from randomization, approval from the medical director and nursing director with a prior protocol explanation) agrees and commits to participating in the study and executing the designated intervention. Having decided which hospitals are eligible, the study will be sent to their own board of ethics committees for careful evaluation and approval.

If this is the case and to proceed with the study, the medical director and nursing director must sign a consent form and all involved nursing staff based on the hospitals’ ethics committee demands. Patients will also be required to sign a document of informed consent to authorize the registration of relevant data regarding their health and medical record. Once each hospital is familiar with the assigned intervention**,** all involved staff will receive instructions for the study implementation and its procedures to be applied in their respective ICUs.

Each intervention will be executed by healthcare staff (nurses and nursing assistants) consecutively according to the allocated intervention in all patients until reaching the study sample in each ICU (150 patients). For ensuring the consistency of the interventions, the total of postural shifts per ICU, in all frequencies, will be recorded daily during a span of 3 months along the course of the intervention by a data collector from patient’s charts from both groups.

A patient will remain in the study from the moment of admission into the ICU until he or she is either transferred to another service or another hospital center, begins walking, or ambulation develops a pressure ulcer or dies.

Each center will designate a leader responsible for the PENFUP phase 2 study procedures in each ICU. Each leader must ensure the complete execution of the project day by day and the accurate application of the assigned intervention in each shift. The healthcare staff assigned to group A (high frequency each 2/ h) will receive automatic text messages by cellphone twice a week to remind the intervention frequency to be applied. Leaders from each center will designate two groups of data collectors to operate based on the informed consent given by patients or their families and will gather information of each intervention day bay day in a register format to be completely archived into the study’s platform, including the number of total daily posture shifts per patient in each ICU.

Each center will receive a set of general initiation instructions in person or via teleconference or internet conference. Each center will also receive the investigators’ manual and questionnaires for collecting data to be included in the study’s platform. The total of postural shifts in 24 h will be registered to maintain proper management of the number of shifts per patient calculated for both high and conventional care. Other variables that will be included contain information of each hospital (the type of hospital, total number of nurses, relation nurse-patients, type of prevention protocols for pressure ulcers, and incidence of a pressure ulcer from the year before the beginning of the study); and patient variables (demographic, health history, health status in ICU, complications in ICU, pressure ulcers and location, repositioning complications and reason of ICU discharge) (Table 1).

**Table 1:** Variables and timeline for inclusion by each ICU. The complete timeline for each ICU is about 3–4 months

Variables and time to collection 1–4 Months by ICU	Variable to be obtained in each ICU				Type of variable by ICU by Intervention and Control			
**Months 1 to 4**	**T1√**	**T2√**	**T3√**	**T4√**	**T1√**	**T2√**	**T3√**	**T4√**
**1-Primary Outcome**	Development of new pressure ulcers in clusters: ▪ Areas defined of injury: sacrum, heels, ankles, elbows, hip, scapula ▪ Others				Rate ulcers per ICU /total control Rate ulcers per ICU/total intervention Median (IQR).			
	Stage of ulcers: I, II, III, IV + o Indeterminate				Total ulcers per stage per ICU per group /150 patients N (%) by cluster			
**1–4 Months by ICU**	**T1√**	**T2√**	**T3√**	**T4√**	**T1√**	**T2√**	**T3√**	**T4√**
**2-Secondary Outcome**	Complications during repositioning				Categorical: Hemodynamic changes, Respiratory Changes, ventilator changes, others.			
	Risk of PU in individuals				Hazard ratio (HR) Date of appearance of the PU (day-month-year) Time to the event (survival analysis)			
**1–4 Months by ICU**	**T1√**	**T2**	**T3**	**T4**	**T1√**	**T2**	**T3**	**T4**
**Variables to be controlled as possible confusion variables**	Old PUs identified upon entry to ICU (presents at the baseline). ▪ Other injuries such as burns, mask injuries, tube injuries, surgical wound dressings In ICU, PUs or dressing site (foam, hydrocolloid)				Total of patients with PUs up-to-baseline/150 patients by cluster (variable to be controlled) N(%)			
**1–4 Months by ICU**	**T1√**	**T2**	**T3**	**T4**	**T1√**	**T2**	**T3**	**T4**
**3-Characteristics of patients [demographic, Health status,**	Age (years), sex (M, F)				Mean (SD), N(%)			
	Body Mass Index (BMI)				Median (interquartile range) or mean (SD)			
	Patients´ Weight at admission				Median (interquartile range, IQR) or mean (SD)			
	Patients´ Height at admission				Median (interquartile range, IQR) or mean (SD)			
	Type of comorbidity to patient admission: Cardiovascular condition, Respiratory condition, Diabetes, Neurological, Malignancy of Carcinoma, Kidney Disease, Peripheral Vascular Disease, Cerebrovascular Accident, Transplant, dermatitis o eczema, documented malnutrition on admission, others.				N(%)			
	Length of hospital stay in ICU (Hours, days) (admission to ICU-Discharge from ICU)				Date: day/month/year Median (interquartile range, IQR) or mean (SD)			
	Patient-days involved in the study (by cluster and				Median (interquartile range, IQR) or mean (SD)			
**1–4 Months by ICU**	**T1√**	**T2√**	**T3√**	**T4√**	**T1√**	**T2√**	**T3√**	**T4√**
**4-Complications of patients in ICU**	Cardiovascular, neurological, sepsis, respiratory, delirium, TEP, musculoskeletal, shock, death				N(%)			
**4-Dates of the possible end of the following in ICU**	First ambulation in ICU Transference to hospital service Hospital transference Death				Day/Month/Year			
**1–4 Months by ICU**	**T1√**	**T2**	**T3**	**T4**	**T1√**	**T2**	**T3**	**T4**
**5-Characteristics of the cluster (or ICU)**	Type of hospital: Public, Private, University, other				N (%)			
	Skin Care Group Preventive care use-report on medical history				N (%)			
	Care guide or policy Institutional Preventive (Yes-No) Name of guideline				N (%)			
	Professional Nurses per shift				Median ó mean (interquartile range IQR)			
	Auxiliares per shift				Median ó mean (interquartile range, IQR)			
**1–4 Months by ICU**	**T1√**	**T2√**	**T3√**	**T4√**	**T1√**	**T2√**	**T3√**	**T4√**
**6-Intervention Characteristic**	Adherence in ICU to intervention				Compliance day by day (yes = 1, no = 2) % compliance (calculated from 80% days stay per patient) ▪ Number of times one patient is mobilized in the intervention ▪ Description of the teams (total number of people who mobilized the patient) ▪ Total number of teams repeating intervention per shift ▪ The number of head and auxiliary nurses per team.			
	Acceptance of the final intervention of the study				Evaluation of the acceptability of the intervention by the personnel			
	Description of usual or conventional care by ICU				Descriptive Individual __or group__ Frequency per shift__ Position variation: yes_ No_ Descriptive.			

If by any chance (including medical assessment or clinical practice setbacks), patient mobilization is decided to be avoided, patients will be excluded from adherence to the intervention (temporarily or definitely), given the pragmatic nature of the study. This information will be registered on the platform. Regular follow-up will be ensured to verify if data is submitted completely and coherently. This will be done by the platform coordinator of PENFUP phase 2. When a pressure ulcer is detected, the nurse in charge of each patient will report it in the individual medical record and will assess the depth of each wound (Fig. 2. SPIRIT figure of study timeline).

### Statistical analysis

All ICUs shall be analyzed according to the branch they were randomized to (Intention to treat) [7, 23]. The incidence of PU will be analyzed considering the time of natural first appearance; that is, within the time-lapse in which the patients are in the study. The two-time variables to be considered for this analysis will be admission into the ICU and the emergence of PUs since the time of admission for each patient. Also, we will include the length of stay in the ICU. The characteristics of the clusters and each individual will be analyzed considering counts (%), means (SD), medians (interquartile range). The percentage of applied interventions (repositioning) by clusters in each group will be computed according to the total of interventions that should be administered in both branches of high frequency and conventional care. The primary analysis will be done at the cluster level (ICU) and the secondary at an individual level (each patient). When analyzing at a cluster level, the total number of PUs will be divided by the total of accumulated days of each patient in each cluster to obtain incidence rates in both groups; this is done based on the permanence of the patients from each cluster. To calculate the incidence rate (IR of 95%), the incidence rate in the groups with a higher frequency of postural changes in group A will be divided by the control groups’ incidence rate of postural changes in group B. The incidence of PU will be analyzed considering the time of natural first appearance; that is, within the time-lapse in which the patients are in the study.

The secondary result includes evaluating the frequency of the severity of PUs (stages I - IV) and will be compared with both groups using an adjusted Chi-square test. For the individual evaluation of the risk of PUs in patients, the first PU to appear in each patient will be considered for calculating the rate of events and time before the event. The hazard ratio (HR) will be computed using Cox proportional-hazard models and its corresponding 95% confidence intervals (CI), adjusted by the standard error by cluster (SE) [23]. The Cox models will be implemented for exploring relations between the main result and the explanatory considering the time before the time of occurrence.

The Hazard Ratio is an estimate that derives from the Cox model, and this is from the rate of risk index between the groups that received a higher frequency rate of postural changes than the groups that received conventional care of postural changes. An adjusted analysis by co-variables will be developed considering the related risk factors in the literature for developing PUs (age and comorbidity). The safety outcomes will be described as the registered events regarding posture shifts in each branch according to their frequency level in interventions A or B, and the association between posture shifts and their compilations will be explored. To detect early differences in the occurrence of events amongst the groups, we planned two interim analyses; once the 33 and 66% of the units are randomized, these internal analyses will be made using the sequential method [23].

## Discussion

The PENFUP-phase 2 project intends to advance the knowledge of efficacy in interventions oriented to preventing PUs in adult patients hospitalized, in this case in the investigation on the efficacy of the application of changes in posture and any complications related to frequent postural changing [24]. The advances from this project will greatly impact direct patient care and the improvement of Preventative Care Guides at a local and word wild scale. This project will also allow a collaborative workflow between hospitals, writing scientific articles, and establishing a network for spreading information based on results that will allow future investigations that promote proper clinical practices.

## Data Availability

Not applicable.

## Abbreviations

PENUP phase 2: Pressure ulcers prevention by nursing, phase 2
ICU: Intensive care units
